# Asymmetric optical camouflage: tuneable reflective colour accompanied by the optical Janus effect

**DOI:** 10.1038/s41377-020-00413-5

**Published:** 2020-10-19

**Authors:** Taehyun Kim, Eui-Sang Yu, Young-Gyu Bae, Jongsu Lee, In Soo Kim, Seok Chung, Seung-Yeol Lee, Yong-Sang Ryu

**Affiliations:** 1 Sensor System Research Center, Korea Institute of Science and Technology, Seoul, 02792 Republic of Korea; 2 Department of Micro/Nano Systems, Korea University, Seoul, 02841 Republic of Korea; 3 School of Electronic and Electrical Engineering, Kyungpook National University, Daegu, 41566 Republic of Korea; 4 Nanophotonics Research Center, Korea Institute of Science and Technology, Seoul, 02792 Republic of Korea; 5 School of Mechanical Engineering, Korea University, Seoul, 02841 Republic of Korea

**Keywords:** Optical sensors, Nanocavities

## Abstract

Going beyond an improved colour gamut, an asymmetric colour contrast, which depends on the viewing direction, and its ability to readily deliver information could create opportunities for a wide range of applications, such as next-generation optical switches, colour displays, and security features in anti-counterfeiting devices. Here, we propose a simple Fabry–Perot etalon architecture capable of generating viewing-direction-sensitive colour contrasts and encrypting pre-inscribed information upon immersion in particular solvents (optical camouflage). Based on the experimental verification of the theoretical modelling, we have discovered a completely new and exotic optical phenomenon involving a tuneable colour switch for viewing-direction-dependent information delivery, which we define as asymmetric optical camouflage.

## Encryption: Nanostructured windows deliver colorful secrets

Applications including anti-counterfeiting measures may benefit from an optical device that encrypts information as viewpoint-dependent colours. Recent studies have shown that embedding metal nanoparticles within transparent materials can produce structural colours that appear differently when seen from the front or back. Yong-Sang Ryu from the Korea Institute of Science and Technology in Seoul and co-workers have now developed a simplified technique for tuning color output from these smart windows. The researchers created optical cavities by sandwiching a porous insulator between a reflecting layer and a film of gold nanoparticles. Dramatic color changes from both viewing directions were achieved by altering the cavity’s effective refractive index using surface modification and liquid infiltration. Engraved messages that were visible in air could be obscured on one side by submersing the windows into specific solvents.

## Introduction

The formation of noble-metal-based nanostructures enables the manipulation of electromagnetic waves, providing controlled transmission, reflection, absorption, and scattering of light^1–6^. In modern optics, a variety of nanoscale materials and their localisation have been examined, as they often lead to novel optical effects^7–11^. Viewing-direction-sensitive information display utilising the optical Janus effect has attracted great attention owing to its dynamic operation scheme, which delivers discriminative information, either in-plane colours/messages^12–14^ or out-of-plane imaging such as holograms^15,16^. Among the schemes, viewing-direction-dependent information delivery across nanostructured windows, which contrasts the popular belief that transparent materials exhibit identical colours/messages when viewed from the front and back^17–22^, has attracted great attention because of its great potential in wide ranges of practical applications. For example, viewing-direction-sensitive asymmetric information displays with broad colour coverage along with real-time colour tuneability can be utilised to realise dynamic colour filters^23–25^, optical switches^26^, data storage devices^27^, anti-counterfeiting devices^28^, and semi-transparent solar cells^29^. However, the integration of a subwavelength geometry in the form of periodic nanostructures often suffers from low throughput associated with the state-of-the-art lithographic techniques necessary for sample fabrication. The issue becomes more complex with the integration of such structures embedded within multiple layers, significantly limiting their application in dynamic and real-time colour tuning. Thus, the development of a new platform overcoming the aforementioned challenges regarding colour tuning is desired for the realisation of advanced optical devices. Furthermore, a simple and cost-effective platform compatible with mass production would be ideal for practical and advanced applications.

**Fig. 1:**
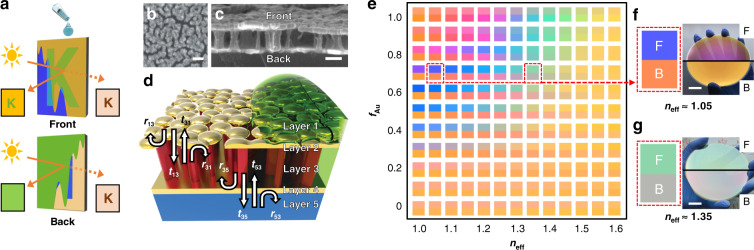
Liquid-permeable BEE for Janus colouration. **a** Conceptual illustration of direction-sensitive asymmetric optical camouflage by the tuneable optical Janus effect. **b**, **c** SEM images of the Bruggeman Au film (**b**, top view) and CYTOP nanopillars (**c**, cross-sectional view) of the BEE. Scale bars, 200 nm. **d** Schematic illustration of light propagation within the liquid-permeable BEE and reflection and transmission coefficients at each interface. **e** Calculated reflective colours of the BEE upon opposite incidences (upper and lower half for reflective colours seen from the frontside and backside) as a function of *f*_Au_ and *n*_eff_ of the dielectric region. **f**, **g** Calculated (left) and experimentally observed (right) reflective colours of BEE under *n*_eff_ ≈ 1.05 **f** and 1.35 **g** seen from the frontside (upper) and backside (bottom). Scale bars, 2 cm

Here, a novel concept utilising a liquid-permeable Fabry–Perot (FP) etalon is proposed to achieve an asymmetric reflective colour contrast dependent on the viewing direction, which is further capable of delivering information. Throughout this work, the mechanisms underpinning the asymmetric colouration in the proposed single unit of a metal−dielectric−metal (MDM) platform as well as a tuneable colour switch for viewing-direction-dependent information display via solvent infiltration are thoroughly analysed using a combined theoretical–experimental approach. Furthermore, a proof-of-concept side-selective anti-counterfeit display is demonstrated based on our state-of-the-art concept of asymmetric optical camouflage (Fig. 1a).

## Results

### Tuneable optical Janus effect via a BEE

First, we describe the design of a transreflective etalon that produces directionally asymmetric reflective colour depending on the incident light direction. Metal nanoparticles within vertical stacks have been demonstrated to play essential roles in viewing-direction-sensitive colour contrast, causing anomalous phase differences in different light reflections^17–19^. As previously reported, a liquid-permeable vertical stack possessing a porous dielectric layer produces different colours based on the refractive index (RI) of the external medium^30^. Colour-tuneable optical Janus effects can be realised by stacking two transreflective metal films of distinct geometries (nanoparticle-like for the top film, flat for the bottom film) separated by a dielectric film with hollow voids (see “Methods” section). Briefly, a representative stack consists of a glass substrate with a 25-nm-thick Au layer, a 150-nm-thick hydrophobic spin-coated CYTOP film, and a 35-nm-thick nanostructured Au layer subsequently self-assembled on a hydrophobic dielectric surface by low-density nucleation^30–33^. O_2_ plasma reactive ion etching (RIE) enables selective etching of the underlying CYTOP film through the nanoapertures of the nanostructured Au film (Fig. 1b), leaving CYTOP nanopillars with hollow air voids (Fig. 1c). This allows infiltration of external media into the dielectric layer. We define the proposed MDM stack as a ‘Bruggeman effective etalon (BEE)’ because the nanomorphologies of both the M and D layers can be regarded as a Bruggeman’s effective medium consisting of metal/air (M) and CYTOP/air (D) composites entangled at the subwavelength scale^34^.

The BEE is composed of five layers (Fig. 1d; layers 1–5 represent air, top Au, dielectric, bottom Au, and glass, respectively), where the top Au and dielectric layers are heterogeneous composite layers of the Bruggeman geometry. Because of the microscopic inhomogeneity of the nanostructures far below the diffraction limit, each layer is regarded as a macroscopically homogeneous medium whose effective optical properties can be derived using Bruggeman’s effective medium theory (see “Methods” section). The total reflection coefficients seen from the frontside and backside (*r*_15_ and *r*_51_, respectively) can be expressed by

1 $$r_{15} = r_{13} + \frac{{t_{13}t_{31}r_{35}\exp (j2\varphi )}}{{1 - r_{31}r_{35}\exp (j2\varphi )}}{\mathrm{and}}\,r_{51} = r_{53} + \frac{{t_{53}t_{35}r_{31}\exp (j2\varphi )}}{{1 - r_{31}r_{35}\exp (j2\varphi )}}$$

where *φ* = *n*_3_*k*_0_*d*_3_cos*θ* is the optical phase difference inside the FP etalon (Fig. S1). This indicates that the reflective colours arise from constructive/destructive interference due to the superposition of multiple waves reflected/transmitted at each interface. By calculating the reflection spectrum using Eq. (1), a simulated colour map (Fig. 1e) was achieved, which displays the correlation between the filling fraction of the top Au layer (*f*_Au_) and the effective RI of the dielectric layer (*n*_eff_). In contrast to viewing-direction-independent colouration in a thin-film cavity^35^ with *f*_Au_ = 1, the BEE possessing nanostructured Au with 0 < *f*_Au_ < 1 obviously induces the optical Janus effect, caused by the asymmetric Au geometry between the metal films (flat for the bottom, nanostructured for the top). Eq. (1) clearly indicates that the reflection coefficients of the first direct reflections (*r*_13_ vs*. r*_53_) as well as the convolution of the transmission coefficients and the internal reflection coefficients for multiple reflections (*t*_13_*t*_31_*r*_35_ vs. *t*_53_*t*_35_*r*_31_) play dominant roles in the direction-sensitive colour display (Fig. S1). This implies that the reflected colour (*r*_15_ vs*. r*_51_) sensitively depends on the geometrical factor (*f*_Au_) as well as the material property (*n*_eff._) of the constituent layer within the BEE stack. Once the structural geometries of the BEE have been established, the overall colour can be further tuned by tailoring *φ*. The colour map shows that the Janus effect is maximised when 0.5 < *f*_Au_ < 0.8, demonstrating that our BEE system is highly advantageous for manipulating colour, as it maintains the optical Janus effect. In addition to the good colour correspondence between the simulation and experimental results, Fig. 1f, g confirm that (i) the generation of predicted colours can be realised by physicochemical control of the BEE geometry and (ii) the proposed platform guarantees colour uniformity and scalability.

### Origin of the optical Janus effect in the BEE architecture

**Fig. 2:**
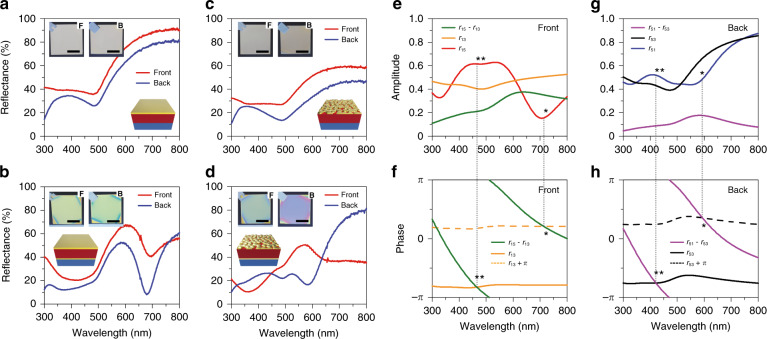
Optical characteristics in different thin film architectures. **a–d** Experimental reflectance spectra of different thin film architectures measured from the frontside (red) and backside (blue) with insets showing photographs and schematics; MD **a**, **c** and MDM structure **b**, **d** with a flat **a**, **b** and a Bruggeman **c**, **d** Au film as the top film. Scale bars, 1 cm. **e–h** Amplitudes **e**, **g** and phases **f**, **h** of reflection coefficients calculated from the film architecture of **d** when seen from the frontside **e**, **f** and backside **g**, **h**

On the basis of a simulation approach to colour contrast and dynamic colour control across viewing directions, we explored the roles of stack architectures and film nanomorphologies in the optical Janus effect. For systematic comparison, we prepared two sets of metal–dielectric (MD) and MDM stacks with different top film geometries. Note that each stack shares a common film thickness and common materials: a 35-nm-thick top Au layer and a 150-nm-thick CYTOP film with/without a 25-nm-thick bottom Au film (Fig. 2a–d). The MD/MDM stacks possessing flat Au on the top exhibit reflection spectra of similar colours and shapes for both sides, with reduced reflectance at the backside due to the ‘substrate effect’^36^ (Fig. 2a, b). Although the asymmetric thickness of the vertically stacked Au layers induces a perceivable colour difference, the results clearly indicate that the flat film by itself is incapable of yielding a colour disparity in the two directions. Additionally, the colour difference between MD (Fig. 2a; silver) and MDM (Fig. 2b; yellow-green) emphasises the role of the FP effect in colour control by shifting the dips in the total reflectance. In contrast to the flat film on top, the optical properties of the Bruggeman stack show the optical Janus effect. More importantly, the colour disparity is intensified in the MDM stack (Fig. 2d) compared with the MD stack (Fig. 2c). Compared with the resonance spectrum within the thin-film FP cavity (Fig. 2b), the resonance of the BEE on the frontside broadened into the longer-wavelength region, whereas that of the backside was blueshifted (Fig. 2d). This phenomenon is attributed to the broadband absorbing Bruggeman film^37^, which causes an overall decrease in reflectivity between 500 and 800 nm (Fig. 2a, c), thereby accelerating the colour contrast across the viewing directions. Taking these results together, the existence of bottom reflectors is essential for perceivable colouration, whereas the optical Janus effect is a result of the top film geometry. While a colour disparity was observed in the reflection mode, the transmission optical properties were found to be identical in both directions because of optical reciprocity (Fig. S2).

To analyse the mechanism underlying the optical Janus effect in the BEE (Fig. 2d), the amplitudes and phases of the reflection coefficients (*r*_15_ and *r*_51_) were calculated. In this process, Bruggeman’s effective medium theory was adopted for optical simulations, where the filling fractions were obtained by image processing and subsequent calculation of the coverages over a large area based on SEM images. The amplitude of *r*_15_ in the calculated spectrum exhibits its maximum reflectance at ~470 nm and minimum reflectance at 720 nm (Fig. 2e), in good agreement with the wavelengths showing phase differences of 0 and *π* between the direct reflection term (*r*_13_) and multiple reflection term (*r*_15_−*r*_13_), respectively (Fig. 2f). In detail, the reflection spectrum of *r*_15_ is a result of interference between *r*_13_ and *r*_15_−*r*_13_, yielding constructive interference at 470 nm (** in Fig. 2e, f) and destructive interference at 720 nm (* in Fig. 2e, f). However, the maximum/minimum reflectances do not completely match the maximum constructive/destructive interferences because of the dispersive property of Au within the visible range. In the case of *r*_51_, the spectral positions of the constructive/destructive interferences were blueshifted to 420 and 600 nm, respectively (Fig. 2g, h), which provides essential clues for the fundamental understanding of the optical Janus effect in the proposed BEE stack, namely, the differences in the two major reflection coefficients (*r*_53_ and *r*_51_−*r*_53_); one is the amplitude of *r*_53_ (orange curve in Fig. 2e vs. black curve in Fig. 2g), and the other is the phase difference of *r*_51_−*r*_53_ between the wavelength spectra of *r*_15_ and *r*_51_. Despite the relative phase similarity of the direct reflection terms under both incidences (*r*_13_ in Fig. 2f vs. *r*_53_ in Fig. 2h), a significant blueshift of the multiple reflection term in *r*_51_ (*r*_51_−*r*_53_; violet curve in Fig. 2h) with respect to *r*_15_ (*r*_15_−*r*_13_; green curve in Fig. 2f) creates a striking colour contrast across the viewing directions. Apparently, the disparity in the range of 500–800 nm intensifies the differences in the multiple reflection terms of the frontside and backside, and the colour contrast becomes complex through the combination of the direct and multiple reflection terms (Fig. S3). In contrast to the reflection spectra of a flat film etalon (Fig. S4), these results highlight the advantage of our BEE architecture for achieving the optical Janus effect.

### Tuneable optical Janus effect by RI modification of the dielectric layer

**Fig. 3:**
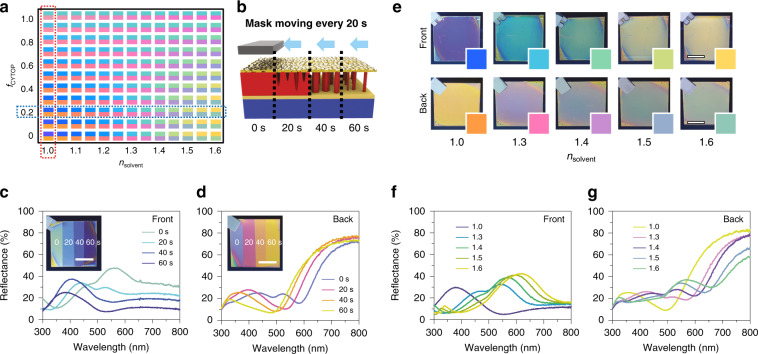
Optical Janus effect with colour tuneability by modifying the effective RI of the dielectric layer. **a** Calculated reflective colours of the BEE upon opposite incidences (upper and lower half seen from the frontside and backside) as a function of *n*_solvent_ and *f*_CYTOP_. **b** Conceptual illustration of controlling the dielectric nanomorphology by moving a mask during RIE. **c**, **d** Experimental reflective optical spectra measured from the frontside **c** and backside **d** with etching durations of 0, 20, 40, and 60 s (insets: photographs of each side). **e**–**g** Photographs of the BEE under media of *n* = 1.0 (air) and *n*_solvent_ = 1.3, 1.4, 1.5, and 1.6 (solvents) (**e**, insets show calculated colours), and their corresponding measured reflective optical spectra **f**, **g**. Scale bars, 1 cm

Having established the origin of the asymmetric colouration, we examined whether modification of the RI of the dielectric layer could provide an additional degree of freedom for modulating the asymmetric optical characteristics of the BEE. As the spectral positions of the FP optical cavity resonance are highly dependent on the RI of the optical spacer region, modification of *n*_eff_ in the dielectric region should induce resonance shifts to the extent that the colour changes in the visible region. Reflective colours under opposite viewing directions were calculated as a function of optical geometric parameters (CYTOP filling fraction; *f*_CYTOP_) and the surrounding RI (*n*_solvent_) prior to the experiments (Fig. 3a). The colour map signifies that smaller values of *f*_CYTOP_ yield more dramatic colour changes with varying *n*_solvent_ without affecting the side-sensitive colour disparity.

Two experiments were designed to demonstrate the role of the dielectric properties of the BEE in the colour control. First, we controlled the *n*_eff_ of the dielectric film utilising spatial RIE to manipulate the dielectric morphology of the BEE. In situ sequential movement of the shadow mask during RIE can produce site-selectively tailored dielectric nanomorphologies (Figs. 3b and S5) and result in etching-time-dependent colouration when viewed from opposite sides; yellow-green (0 s), light-blue (20 s), blue (40 s), and bluish-violet (60 s) were observed on the frontside, whereas violet (0 s), pink (20 s), orange (40 s), and yellow (60 s) were monitored on the backside (Fig. 3c, d), in good agreement with the simulated reflective colours (red dotted box in Fig. 3a). As more air (*n* = 1.0) enters the voids of the dielectric region with longer etching, the decreased *n*_eff_ of the dielectric region (ideally, 1 < *n*_eff_ < 1.34) induces a spectral blueshift in both viewing directions, which signifies the effect of physical modification of the dielectric regions. Second, chemical modification of the dielectric layer was also examined by infiltrating transparent solvents with various RIs into the porous dielectric layer. For a BEE with an etching duration of 60 s (*f*_Au_ = 0.65, *f*_CYTOP_ = 0.20), the reflective colours exhibited asymmetric F/B colour contrasts of bluish-violet/yellow (air; *n* = 1.0), cyan/pink (*n*_solvent_ = 1.3), green/violet (*n*_solvent_ = 1.4), yellow-green/blue-green (*n*_solvent_ = 1.5), and yellow/green (*n*_solvent_ = 1.6) in both experiments (Fig. 3e) and simulations (insets in Fig. 3e). The experimental reflectance spectra clearly demonstrate that the capillary force drives solvents into the prearranged air voids, thereby modifying the *n*_eff_ of the dielectric region and ultimately causing dramatic colour changes in both viewing directions (Fig. 3f, g). Though they are hardly recognisable with the naked eye, transmission colour changes were also monitored that showed great correspondence with the theoretical modelling (Fig. S6). Scalable and uniform colouration display with active colour tuning through dielectric modification proves the versatility of our BEE platform, which is among the key prerequisites for practical applications (Figs. S7 and S8). This demonstrates that tuneable colouration can be achieved via manipulation of the dielectric properties while maintaining the optical Janus effect.

### Symmetric message encryption/decryption by dielectric tuning

**Fig. 4:**
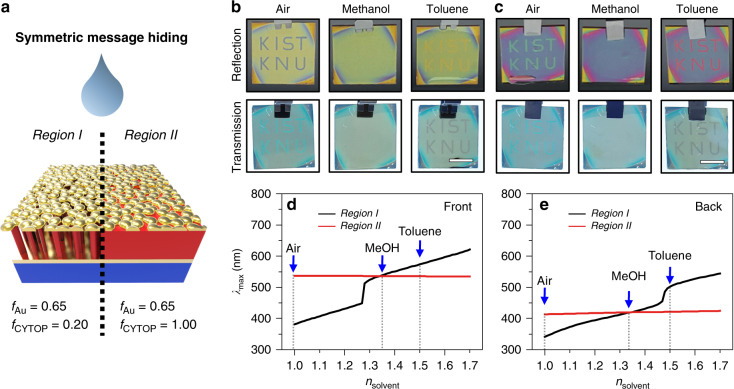
Site-selectively etched BEE for symmetric message hiding. **a** Schematic illustration showing different *f*_CYTOP_ values for symmetric message hiding. **b**, **c** Photographs of the site-selectively etched BEE (*Region I* for the message and *Region II* for the background) demonstrating symmetric message hiding in reflection (upper row) and transmission (lower row) modes seen from the frontside **b** and backside **c** under air, methanol, and toluene. Scale bars, 1 cm. **d**, **e** Calculated spectral positions of the maximum reflectance in *Regions I* (black line) and *II* (red line) seen from the frontside **d** and backside **e** as a function of *n*_solvent_

The proposed BEE can be applied to photonic message encryption. A message was pre-encoded on the BEE surface through site-selective RIE using a message-open shadow mask, creating two different regions of distinct dielectric nanomorphologies: *f*_Au_ = 0.65 and *f*_CYTOP_ = 0.20 for *Region I* and *f*_Au_ = 0.65 and *f*_CYTOP_ = 1.00 for *Region II* (Figs. 4a and S9). When immersed in index-matching solvents (methanol; *n* = 1.34), the optical properties of the encoded messages in *Region I* were designed such that their colours matched those of the background in *Region II* (Fig. 4a). As a result, the encoded message became indistinguishable in both reflection and transmission modes (optical camouflage) because the background/message regions were optically identical under index-matching conditions (methanol; *n* = 1.34). In non-index-matching solvents (toluene; *n* = 1.49, Fig. 4b, c, and Supplementary video 1), however, the encoded messages became distinguishable. Since the colour contrast is determined dominantly by the difference between the *n*_eff_ of the message region (given by the difference between the RIs of CYTOP and the medium) and the background region (fixed at *n* = 1.34), the larger difference in *n*_eff_ across the boundary in air leads to a larger colour contrast than in toluene. For a more systematic analysis, the reflection spectra were simulated, and the spectral positions of the local maximum reflectance (*λ*_max_) were plotted as a function of *n*_solvent_ (Figs. 4d, e, and S10). As the identical *f*_Au_ values in *Regions I* and *II* guarantee identical intersection of *λ*_max_ for both sides (front and back) at *n*_solvent_ = 1.34, direction-insensitive optical camouflage with an index-matching solvent is capable of information encryption, regardless of the viewing direction as well as observation mode.

### Asymmetric information encryption by optical camouflage

**Fig. 5:**
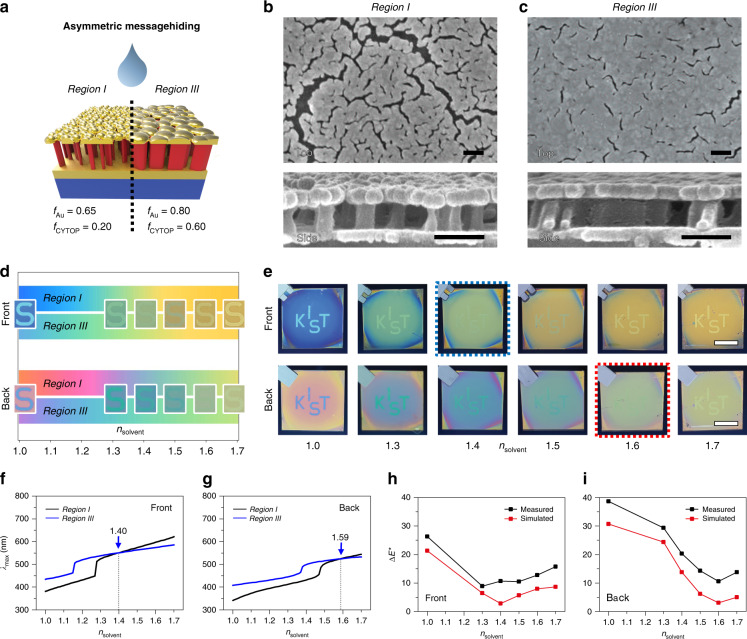
BEE for asymmetric message hiding. **a** Schematic illustration showing different *f*_Au_ and *f*_CYTOP_ values for viewing-direction-sensitive message hiding. **b**, **c** Top-view (upper) and cross-sectional view (lower) SEM images of *Regions I* **b** and *III* **c**. Scale bars, 200 nm. **d** Calculated reflective colour gradation in each region seen from both sides (insets show enlarged photographs of **e**). **e** Photographs of the solvent-immersed BEE (*Region I* for the background and *Region III* for the message) demonstrating asymmetric message hiding seen from the frontside (upper) and backside (lower) under media of *n* = 1.0 (air) and *n*_solvent_ = 1.3, 1.4, 1.5, 1.6, and 1.7 (solvents). Scale bars, 1 cm. **f**, **g** Calculated spectral positions of the maximum reflectance in *Regions I* (black line) and *III* (blue line) seen from the frontside **f** and backside **g** as a function of *n*_solvent_ = 1.0–1.7. **h**, **i** ΔE^*^ between *Regions I* and *III* as a function of *n*_solvent_ obtained from calculated (red line) and measured (black line) reflectance spectra seen from the frontside **h** and backside **i**

In contrast to symmetric message encryption through optical camouflage, viewing-direction-sensitive asymmetric information encryption/decryption was examined by tuning the geometries of the constituent films, such as *f*_Au_ and *f*_CYTOP_. Inspired by the colour tuneability that depends on the physical properties of the layers comprising the BEE (Fig. 1e), we engineered both *f*_Au_ and *f*_CYTOP_ by modifying the surface hydrophobicity of the dielectric surface (*Region III*) through O_2_ plasma surface treatment prior to the top Au deposition. Since the self-assembled top Au nanomorphology is highly dependent on the surface energy, site-selective control of the hydrophobicity of the CYTOP surface yields distinct values of *f*_Au_ during the Au deposition process. The sequential RIE modifies *f*_CYTOP_, tailoring the dielectric geometry with various proportions of hollow voids (Figs. 5a and S9). Comparing the geometric properties of *Region I* (Fig. 5a, b, *f*_Au_ = 0.65, *f*_CYTOP_ = 0.20), the experimental surface modification and subsequent RIE process successfully modifies both the top Au and dielectric nanomorphologies of *Region III* in a less perforated (*f*_Au_ = 0.80) and less etched (*f*_CYTOP_ = 0.60) manner (Fig. 5a, c). Based on the experimentally achieved geometric parameters, the reflective colours and spectra on both sides of *Regions I* and *III* were simulated with respect to the surrounding RI (Figs. 5d and S10). The simulated results suggest that the reflective colours in the two different regions are analogous around *n*_solvent_ = 1.4 and 1.6 for the frontside and backside, respectively. To experimentally verify the simulated results, we prepared a message-encoded BEE sample through selective surface modification and compared the colours of each region (*Region III* for message, *Region I* for background) upon infiltrating various solvents. As seen from both viewing directions, the message was clearly distinguishable under an atmospheric environment, showing great colour disparities across the two regions (Fig. 5e; *n* = 1.0). However, when immersed in standard RI liquids, the frontside reflective optical messages were barely distinguishable around *n*_solvent_ = 1.4 (blue dotted box in Fig. 5e), whereas they were indistinguishable at *n*_solvent_ = 1.6 on the opposite side (red dotted box in Fig. 5e and Supplementary video 2). The viewing-direction-sensitive information encryption through optical camouflage was qualitatively analysed by *λ*_max_ (Fig. 5f, g) and the colour differences (ΔE^*^) (Fig. 5h, i). Note that the left and right of the backside photographs were flipped for ease of presentation.

The calculated *λ*_max_ as a function of *n*_solvent_ obviously confirms that the reflection peaks intersect upon exposure to different solvents, such as under *n*_solvent_ = 1.40 and 1.59 for the frontside and backside, respectively (Figs. 5f, g and S10). Although the calculated colour maps (Fig. 5d) showed great correspondence with the experimental observations (Fig. 5e), a more detailed analysis should be conducted because identical spectral wavelengths of the maximum reflectance do not always guarantee cognitive colour similarities across two regions. In this respect, the colour differences (ΔE^*^), which can quantify the cognitive similarities between two different colours^38^, were calculated from each measured/simulated spectra and marked in the CIE 1931 map (Figs. S11 and S12). According to the calculations, ΔE^*^ became minimised under *n*_solvent_ = 1.4 and 1.6 when viewed from the frontside and backside, respectively (red lines in Fig. 5h, i). Because the minimised ΔE^*^ values imply that the reflection colours from the two regions become barely distinguishable, the analysis evidently supports the exotic optical phenomenon of viewing-direction-sensitive information encryption through optical camouflage. In contrast to the side-selective message encryption, the colour contrast across the two regions remained when observed in transmission mode (Fig. S13 for transmission mode). In addition, owing to the low angular dependency, the asymmetric message maintained its invisibility with variation of the viewing angle to a reasonable extent (Figs. S14, S15 and Supplementary video 3) and even under diffuse illumination such as natural sunlight (Fig. S16, Supplementary videos 4 and 5), and it can be further improved in combination with strategies that reduce the angular dependency^39,40^. These results suggest that manipulation of film geometries (*f*_Au_ and *f*_CYTOP_) enables colour contrast display for information encoding and allows viewing-direction-sensitive information delivery through optical camouflage.

## Discussion

Although the novel optical Janus effect opens up a new avenue for asymmetric colour generation, the limited size/shape of nanoparticles embedded within multiple films has prohibited its application in dynamically tuneable colour displays. Our proposed transreflective BEE platform exhibits asymmetric reflective colour display along with colour tuneability with infiltration of solvents. We discovered that precise control of the physicochemical properties of the films (*f*_Au_ and *f*_CYTOP_) comprising the BEE stack leads to (i) enhanced colour contrast with respect to the viewing direction and (ii) superior optical resonance shifts for independent colour control upon *n*_eff_ modulation via liquid infiltration. Owing to the low angular dependency arising from the thin dielectric thickness and resonance broadening by the random Au nanomorphology, the proposed BEE retains its asymmetric message-hiding characteristics at wide viewing angles or under diffuse illumination, such as in natural sunlight conditions. As a result, compared with previously demonstrated discriminative information delivery based on photonic crystals^13^, our proposed BEE is highly advantageous in terms of the reasonable maintenance of its optical properties under off-axis or diffuse illumination, which is essential for real-world applications under various illumination conditions. Taking a step further, we developed a spatially selective tailoring process for modification of the dielectric nanomorphology to engrave informative messages on the BEE surface, which confirmed the state-of-the-art concept of a chromatically switchable phenomenon accompanied by viewing-direction-sensitive optical camouflage under immersion in different solvents. Moving forward, bidirectional display of two different tuneable messages/images appears to be a tangible goal in the next step to enable a wider range of photonic applications, including dynamic/informative colour filters, smart windows, optical switches, double-sided colour displays, optical data storage devices, and anti-counterfeiting devices. Moreover, beyond presenting a tuneable Janus effect by tuning the RIs of dielectric films, our universal scheme for manipulating optical properties through an MDM stack can be implemented by adopting specialised nanomaterials, such as temperature-sensitive^24^ or humidity-sensitive materials^25^ and phase-change materials^41,42^, which potentially open up tremendous opportunities for substantive optical device applications. Furthermore, as the proposed fabrication process for our BEE is compatible with large-area substrates, the design can be applied to optical-sensing platforms, including surface-enhanced Raman spectroscopy^43^, localised surface plasmon resonance^44^, and surface-enhanced infrared absorption^45^.

## Materials and methods

### Fabrication process of the BEE

A glass substrate (2.5 cm × 2.5 cm) was cleaned with piranha solution (30% H_2_O_2_/70% H_2_SO_4_ (v/v)) at 120 °C for 15 min and rinsed in deionised water. Then, a 25-nm-thick Au film was deposited on the substrate using an e-beam evaporator (ei-5k, ULVAC). After preparing a 4.5 wt% CYTOP solution by mixing 9 wt% CYTOP solution (CTL-809M, AGC Chemical) with a solvent (CT-Solv. 180, AGC Chemical) in a 1:1 ratio, the solution was spin-coated on the Au surface at 3000 rpm for 45 s and thermally annealed on a hotplate at 80 °C for 60 min. Subsequently, a top Au layer was deposited by a thermal evaporator (SHE-68-350D, Samhan Vacuum Development) under evaporation conditions of a vacuum level of 10^-6^ Torr and an evaporation rate of 0.3 Å/s, which promotes low-density nucleation for self-assembly. Finally, O_2_ plasma RIE (100 mTorr, plasma generation frequency of 13.56 MHz, RF power of 150 W, and O_2_ gas flow rate of 100 sccm) was carried out using a reactive ion etcher (RIE 80 plus, Oxford Instrument) to tailor the dielectric layers into nanopillars. Note that several fabrication conditions were exceptionally modified for special cases. For example, the evaporation rate of the top Au layer was adjusted to 3.5 Å/s for an optical etalon with a flat Au film (Fig. 2a, b). In particular, for ‘symmetric message hiding’ (Fig. 4), a shadow mask was employed for site-selective etching of the dielectric layer during RIE. In the case of ‘asymmetric message hiding’ (Fig. 5), site-selective O_2_ plasma treatment (CUTE plasma system, Femto Science; 500 mTorr pressure, plasma generation frequency of 50 kHz, RF power of 70 W, and O_2_ gas flow rate of 30 sccm) was performed for 5 s prior to top Au deposition with a shadow mask on the CYTOP surface.

### Optical measurement

Under normal incidence white light (DH-MINI, Ocean optics), reflectance, and transmittance signals were detected using ultraviolet–visible spectroscopy (USB4000-UV–VIS, Ocean optics). For reflection measurement, the optical signals were transmitted and received through a reflection probe (R400-7-SR, Ocean optics) and normalised using an aluminium standard mirror (STAN-SSH, Ocean optics). The transmittance was measured with transmission probes (QP 300-1-SR, Ocean optics) and UV/VIS collimating lenses (74-UV, Ocean optics). For modification of the surrounding RI, standard RI solvents (RI liquids, Cargille) were used with RIs of *n*_solvent_ = 1.3, 1.4, 1.5, 1.6, and 1.7.

### Effective optical properties calculated using Bruggeman’s effective medium theory

For simulation, Bruggeman’s effective medium theory was used. The effective optical properties of subwavelength composite layers, such as the top Au film and etched CYTOP layer, were estimated using Bruggeman’s effective medium theory. The effective permittivity of a medium (*ε*_eff_) is given by

$$f_1\frac{{\varepsilon _1 - \varepsilon _{{\rm {eff}}}}}{{\varepsilon _1 + \kappa \varepsilon _{{\rm {eff}}}}} + \left( {1 - f_1} \right)\frac{{\varepsilon _2 - \varepsilon _{{\rm {eff}}}}}{{\varepsilon _2 + \kappa \varepsilon _{{\rm {eff}}}}} = 0$$

where *κ*, *f*, and *ε* are the screening parameter, filling fractions, and permittivities of two different materials. While *ε*_eff_ of the top Au film (layer 2) was obtained by assuming *ε*_1_ = *ε*_Au_, *ε*_2_ = *ε*_medium_, and *f*_1_ = *f*_Au_, the same procedure could also be performed to calculate the *ε*_eff_ of the dielectric region (layer 3) from *ε*_1_ = *ε*_CYTOP_, *ε*_2_ = *ε*_medium_, and *f*_1_ = *f*_CYTOP_, in which *ε*_Au_, *ε*_medium_, and *ε*_CYTOP_ are the permittivities of Au^46^, the surrounding medium (such as air or liquid), and CYTOP. Note that a screening parameter of *κ* = 1 was used because the layers can be regarded as two-dimensional structures^47^.

### Scanning electron microscope (SEM) measurement and data analysis

After depositing a 2-nm-thick platinum layer on the samples using an ion sputter coater (E-1045, Hitachi), high-resolution morphology observations were performed using an SEM (Nova NanoSEM 200, FEI) equipped with a through-lens detector (10 kV, 140 μA, working distance: 5 mm). To evaluate the filling fractions of Au and CYTOP, the SEM images were analysed using image-processing software (ImageJ, National Institutes of Health).

## Supplementary information

Supplementary Information for Asymmetric Optical Camouflage: Tuneable reflective colour accompanied by the optical Janus effect

Site-selectively etched BEE for symmetric message encryption

BEE for asymmetric message encryption

Angle-dependent colour variations in asymmetric message hiding BEE

Asymmetric message hiding under natural sunlight

Asymmetric message hiding under natural sunlight illumination

## Data Availability

The data that support the findings of this study are available from the corresponding author upon reasonable request.

## References

[1] Yu NF (2011). Light propagation with phase discontinuities: generalized laws of reflection and refraction. Science.

[2] Ni XJ (2012). Broadband light bending with plasmonic nanoantennas. Science.

[3] Zheludev NI, Kivshar YS (2012). From metamaterials to metadevices. Nat. Mater..

[4] Chen XZ (2012). Dual-polarity plasmonic metalens for visible light. Nat. Commun..

[5] Monticone F, Estakhri NM, Alù A (2013). Full control of nanoscale optical transmission with a composite metascreen. Phys. Rev. Lett..

[6] Kristensen A (2017). Plasmonic colour generation. Nat. Rev. Mater..

[7] Sounas DL, Alù A (2017). Non-reciprocal photonics based on time modulation. Nat. Photonics.

[8] Tsakmakidis KL (2017). Breaking Lorentz reciprocity to overcome the time-bandwidth limit in physics and engineering. Science.

[9] Xu T (2010). Plasmonic nanoresonators for high-resolution colour filtering and spectral imaging. Nat. Commun..

[10] Miyata M, Hatada H, Takahara J (2016). Full-color subwavelength printing with gap-plasmonic optical antennas. Nano Lett..

[11] Bao YJ (2019). Full-colour nanoprint-hologram synchronous metasurface with arbitrary hue-saturation-brightness control. Light Sci. Appl..

[12] Yu P (2018). Dynamic Janus metasurfaces in the visible spectral region. Nano Lett..

[13] Fan W (2019). Iridescence-controlled and flexibly tunable retroreflective structural color film for smart displays. Sci. Adv..

[14] Gao YS (2018). Lead halide perovskite nanostructures for dynamic color display. ACS Nano.

[15] Chen K (2020). Directional Janus metasurface. Adv. Mater..

[16] Hu YQ (2020). Trichromatic and tripolarization-channel holography with noninterleaved dielectric metasurface. Nano Lett..

[17] England GT (2017). The optical Janus effect: asymmetric structural color reflection materials. Adv. Mater..

[18] Saito K, Tatsuma T (2015). Asymmetric three-way plasmonic color routers. Adv. Opt. Mater..

[19] Saito K, Tatsuma T (2016). Control of asymmetric scattering behavior of plasmonic nanoparticle ensembles. ACS Photonics.

[20] Duempelmann L (2016). Four-fold color filter based on plasmonic phase retarder. ACS Photonics.

[21] Ye F, Burns MJ, Naughton MJ (2014). Structured metal thin film as an asymmetric color filter: the forward and reverse plasmonic halos. Sci. Rep..

[22] Butun S, Aydin K (2015). Asymmetric light absorption and reflection in freestanding nanostructured metallic membranes. ACS Photonics.

[23] Wang GP (2016). Mechanical chameleon through dynamic real-time plasmonic tuning. ACS Nano.

[24] Sorrell CD, Carter MCD, Serpe MJ (2011). Color tunable poly (*N*-isopropylacrylamide)-*co*-acrylic acid microgel–Au hybrid assemblies. Adv. Funct. Mater..

[25] Kwon H, Kim S (2015). Chemically tunable, biocompatible, and cost-effective metal–insulator–metal resonators using silk protein and ultrathin silver films. ACS Photonics.

[26] Chai Z (2016). On-chip optical switch based on plasmon–photon hybrid nanostructure-coated multicomponent nanocomposite. Adv. Opt. Mater..

[27] Zijlstra P, Chon JWM, Gu M (2009). Five-dimensional optical recording mediated by surface plasmons in gold nanorods. Nature.

[28] Yoon B (2013). Recent functional material based approaches to prevent and detect counterfeiting. J. Mater. Chem. C.

[29] Atwater HA, Polman A (2010). Plasmonics for improved photovoltaic devices. Nat. Mater..

[30] Yu ES (2018). Highly sensitive color tunablility by scalable nanomorphology of a dielectric layer in liquid-permeable metal–insulator–metal structure. ACS Appl. Mater. Interfaces.

[31] Jeffers G, Dubson MA, Duxbury PM (1994). Island-to-percolation transition during growth of metal films. J. Appl. Phys..

[32] Leosson K (2013). Ultra-thin gold films on transparent polymers. Nanophotonics.

[33] Dostálek J, Kasry A, Knoll W (2007). Long range surface plasmons for observation of biomolecular binding events at metallic surfaces. Plasmonics.

[34] Cai, W. S. & Shalaev, V. *Optical Metamaterials: Fundamentals and Applications* (Springer, New York, 2010).

[35] Li ZY, Butun S, Aydin K (2015). Large-area, lithography-free super absorbers and color filters at visible frequencies using ultrathin metallic films. ACS Photonics.

[36] Barybin A, Shapovalov V (2010). Substrate effect on the optical reflectance and transmittance of thin-film structures. Int. J. Opt..

[37] Xue JC (2015). Scalable, full-colour and controllable chromotropic plasmonic printing. Nat. Commun..

[38] Mokrzycki WS, Tatol M (2011). Colour difference ∆*E*—a survey. Mach. Graph. Vis. Int. J..

[39] Park CS (2015). Omnidirectional color filters capitalizing on a nano-resonator of Ag–TiO_2_–Ag integrated with a phase compensating dielectric overlay. Sci. Rep..

[40] Lee KT (2014). Strong resonance effect in a lossy medium-based optical cavity for angle robust spectrum filters. Adv. Mater..

[41] Hosseini P, Wright CD, Bhaskaran H (2014). An optoelectronic framework enabled by low-dimensional phase-change films. Nature.

[42] Lee SY (2017). Holographic image generation with a thin-film resonance caused by chalcogenide phase-change material. Sci. Rep..

[43] Ding SY (2016). Nanostructure-based plasmon-enhanced Raman spectroscopy for surface analysis of materials. Nat. Rev. Mater..

[44] Willets KA, van Duyne RP (2007). Localized surface plasmon resonance spectroscopy and sensing. Annu. Rev. Phys. Chem..

[45] Adato R, Aksu S, Altug H (2015). Engineering mid-infrared nanoantennas for surface enhanced infrared absorption spectroscopy. Mater. Today.

[46] Rakić AD (1998). Optical properties of metallic films for vertical-cavity optoelectronic devices. Appl. Opt..

[47] Bruggemann DAG (1935). Berechnung verschiedener physikalischer konstanten von heterogen substanzen. Ann. Phys..

