# Supplementary material for: Asymmetric optical camouflage: tuneable reflective colour accompanied by the optical Janus effect
Source: Light Sci Appl. 2020 Oct 19;9:175. doi: 10.1038/s41377-020-00413-5 (PMC7569085; doi:10.1038/s41377-020-00413-5)
Supplement: Supplementary file 1 — Supplementary Information for Asymmetric Optical Camouflage: Tuneable reflective colour accompanied by the optical Janus effect [file 41377_2020_413_MOESM1_ESM.docx]

Supplementary Information for

**Asymmetric Optical Camouflage: Tuneable reflective colour accompanied by the optical Janus effect**

Taehyun Kim^1,2,†^, Eui-Sang Yu^1,†^, Young-Gyu Bae^3,†^, Jongsu Lee^1^, In Soo Kim^4^,

Seok Chung^2,5^, Seung-Yeol Lee^3,*^, and Yong-Sang Ryu^1,*^

^1^*Sensor System Research Center, Korea Institute of Science and Technology, Seoul 02792, Republic of Korea*

^2^*Department of Micro/Nano Systems, Korea University,* *02841, Seoul, Republic of Korea*

^3^*School of Electronics Engineering, Kyungpook National University, Daegu 41566, Republic of Korea*

*^4^Nanophotonics Research Center, Korea Institute of Science and Technology, Seoul 02792, Republic of Korea*

^5^*School of Mechanical Engineering, Korea University,* *02841, Seoul, Republic of Korea*

^†^*These authors contributed equally*.

**Correspondence and requests for materials should be addressed to*

*S.-Y.L. (seungyeol@knu.ac.kr) or Y.-S.R. (ysryu82@kist.re.kr)*.

**Supplementary figures**


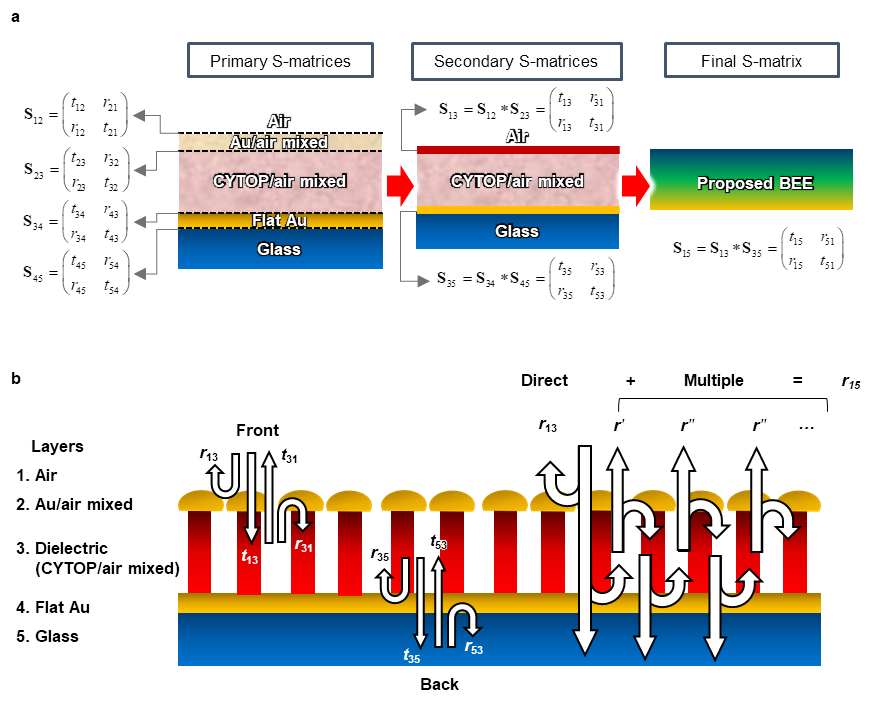


**Fig. S1 Calculation of light-direction-dependent transmission and reflectance on Bruggeman effective etalon (BEE) using Redheffer’s star-product. a** The schematic illustrates the mechanism to obtain the transmission (*t_ik_*: transmission light from *i* to *k*) and reflection (*r_ik_*) coefficients of the proposed BEE structure using Redheffer’s star-product of scattering matrices. The primary scattering matrices (**S**-matrices) at the layer interface between the two layers (layer numbers *i* and *j*) are defined as follows,

$\mathbf{S}_{ij}=\left(\begin{matrix} t_{ij} & r_{ji} \\ r_{ij} & t_{ji} \end{matrix}\right)=\frac{1}{n_{i}+n_{j}}\left(\begin{matrix} 2n_{i} & n_{j}-n_{i} \\ n_{i}-n_{j} & 2n_{j} \end{matrix}\right)$ for *j* = *i* + 1.

where *n*, *r*, and *t* indicate the effective refractive index (RI) of the layer and the complex coefficients of reflection and transmission, respectively. The Redheffer star-product can be used to calculate a cascaded interconnection of two scattering matrices in optics as follows,

*d_j_* and *θ* denote the distance between two scattering matrix systems and the incident angle of light, respectively. To obtain the total transmission and reflection coefficients across the entire structure of BEE, Redheffer’s star-product calculation was employed as follows^1^,

$\left(\mathbf{S}_{12}*\mathbf{S}_{23}\right)*\left(\mathbf{S}_{34}*\mathbf{S}_{45}\right)=\mathbf{S}_{13}*\mathbf{S}_{35}=\left(\begin{matrix} t_{15} & r_{51} \\ r_{15} & t_{51} \end{matrix}\right)=\mathbf{S}_{15}$,

reducing four different interfacial **S**-matrices (**S**_12_, **S**_23_, **S**_34_, and **S**_45_ between five layers of the BEE) into a single **S**_15_. Because the sequence of calculation does not affect the final results, we first performed calculation for the top Au and bottom Au layers, then finally evaluated the dielectric layer.

**b** Schematic of BEE and optical coefficients consisting of S-matrix. The total reflection coefficient at the frontside is defined as

,

where the first- (*r*_13_) and the second terms (*r*_15_ – *r*_13_) denote direct reflection at the first stage and multiple reflection within the metallic layers, respectively.

**
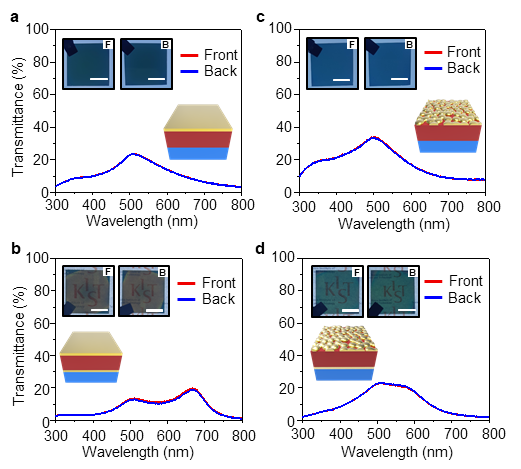
**

**Fig. S2 Stack of thin films with different architectures.** **a-d** Measured transmittance spectra of different architectures with schematics and photographs (scale bars, 1 cm); metal–dielectric structure (**a**, **c**) and metal-dielectric-metal structure (**b**, **d**) consisting of flat- (**a**, **b**) and Bruggeman (**c**, **d**) Au films on top (‘F’ and ‘B’ denote the frontside and backside, respectively).

**
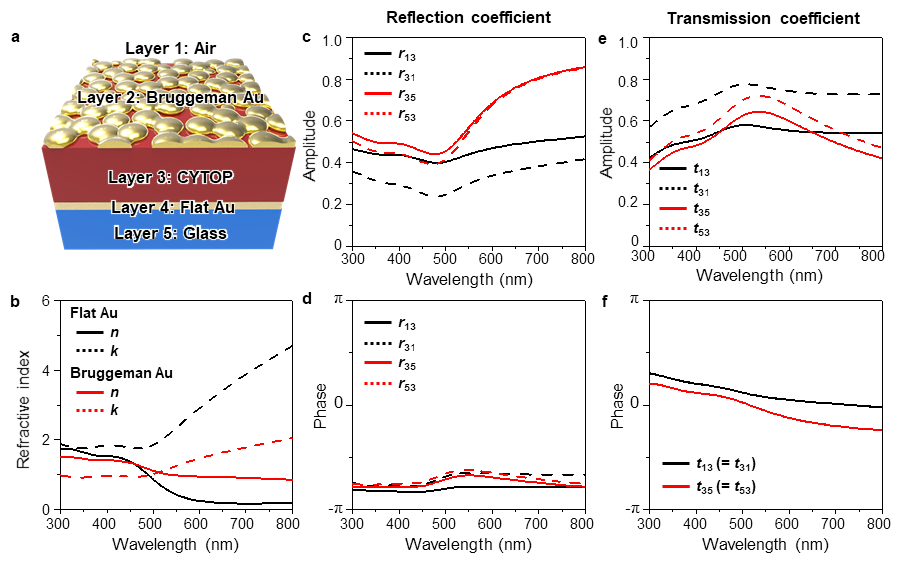
**

**Fig. S3 Optical coefficients of BEE.** To identify the effect of film geometry on the total reflection coefficient of a BEE, we calculated the complex refractive indices of Au in accordance with its film geometries. Using this result, reflection (*r_ik_*)- and transmission (*t_ik_*) coefficients were calculated for incident light direction from layer *i* to layer *k*. **a** Schematic illustration of BEE with layer information. **b** Calculated refractive indices of flat- and Bruggeman-structured (*f*_Au_ = 0.65) Au film. In the case of Bruggeman Au, its metallic property is diminished because its nanostructure is filled with air **c**, **d** Amplitude (**c**) and phase (**d**) of reflection coefficients for the top Bruggeman- (black) and bottom flat (red) Au films (**solid line**; incident light direction from the frontside to the backside, **dashed line**; vice versa). The difference between absolute values of Bruggeman- and flat Au film is observed especially in the long wavelength range (500 – 800 nm). Meanwhile, the difference in phases is barely noticeable (**d**). **e**, **f** Amplitude of transmission coefficients (**e**) also shows disparity between film geometries, which occurs with an intensity change in multiple terms (*r*_15_ - *r*_13_ and *r*_51_ - *r*_53_). Because the destructive interference shift is primarily affected by the phase of the transmission coefficient, the disparity between *t*_13_ and *t*_35_ is one of the key factors in the Janus effect (**f**).

**
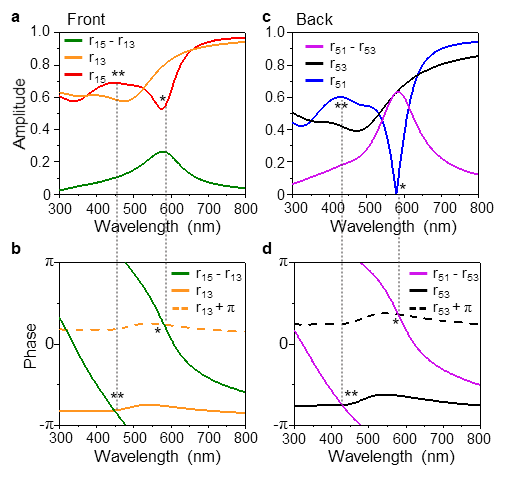
**

**Fig. S4 Optical properties of a flat film etalon. a-d** Calculated amplitudes (**a**, **c**) and phases (**b**, **d**) of the reflection coefficients from the etalon with flat Au film (**a**, **b**: frontside, **c**, **d**: backside). The simulated amplitude spectra of *r*_15_ achieved from either side show great correspondence with the measured spectra (**main Fig. 2b**) as they share identical constructive- (** in **Figs. 2e** and **2f**) and destructive interferences (* in **Figs. 2e** and **2f**). Unlike the BEE, moreover, broadband absorption seen from the frontside is barely able to be measured. This clearly implies that Bruggeman Au geometry yields broadband absorption in the long wavelength range (500–800 nm).


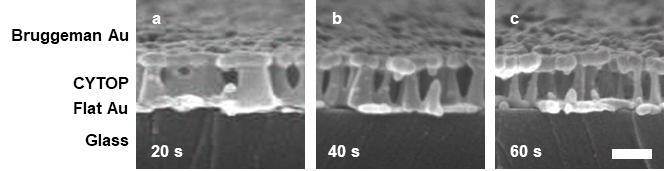


**Fig. S5 Tailoring dielectric nanomorphology of BEE through RIE. a-c** Cross-sectional SEM images of BEE with reactive ion etching (RIE) durations of 20 s (**a**), 40 s (**b**), and 60 s (**c**). Scale bar, 100 nm.


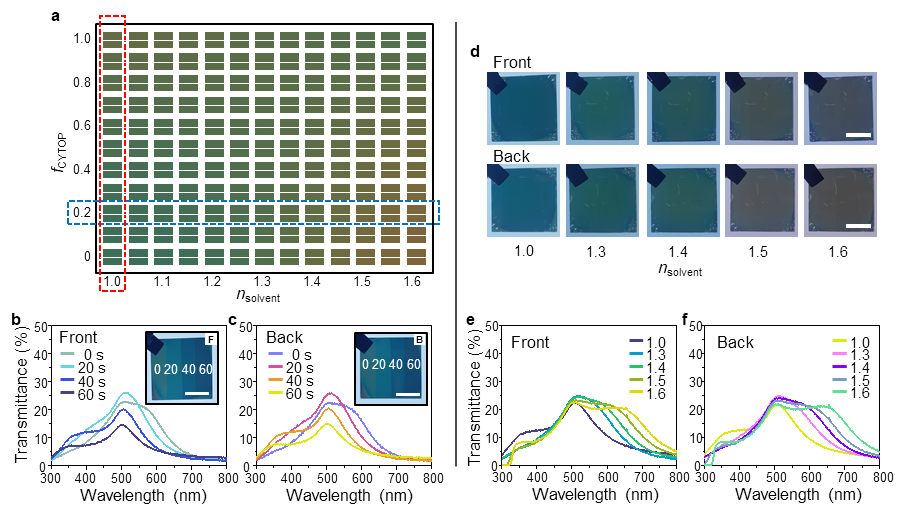


**Fig. S6 Transmissive colour tuneability by modifying effective RI of dielectric layer. a** Simulated transmissive colour map (upper half of each box for frontside and lower half for backside) as a function of *n*_solvent_ and *f*_CYTOP_. For example, the red and blue dotted boxes each denote the expected tuneable colouration controlled by a specific solvent immersion (*n*_solvent_) and etching duration (*f*_CYTOP_), respectively. **b**, **c** Experimental transmissive optical spectra measured from the frontside (**b**) and the backside (**c**) with etching durations of 0 s, 20 s, 40 s, and 60 s (insets: photographs of the sample). **d**-**f** Photographs of the BBE covered with media of *n* = 1.0 (air), *n*_solvent_ = 1.3, 1.4, 1.5, and 1.6 (solvents) (**d**) and their corresponding transmissive optical spectra (**e**, **f**). Note that the transmittance peak at 500 nm arises from the intrinsic optical properties of Au. The results show that the colour contrast across the viewing direction is barely perceivable in transmissive mode. Scale bars, 1 cm.

**
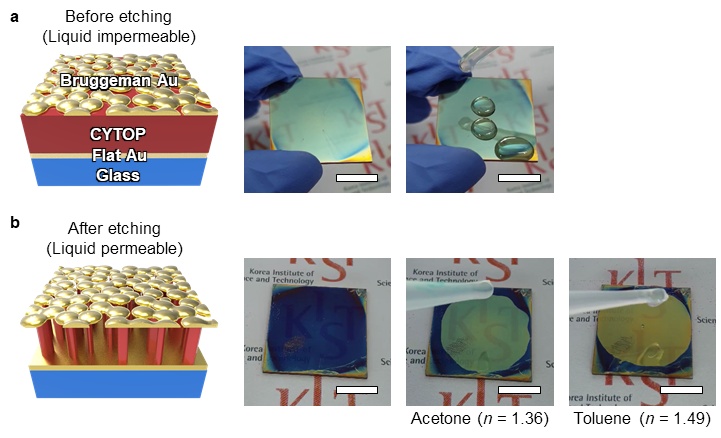
**

**Fig. S7 Liquid permeability and colour tuneability. a**, **b** Schematics and photographs of the liquid impermeable- (**a**) and permeable BEE (**b**). Tailoring the dielectric nanomorphology through RIE (50 s) allows the outer mediums to infiltrate into the hollow voids of the dielectric layer, thereby identifying the medium by its distinct colour (air for dark-blue; *n* = 1.00, acetone for green; *n*_solvent_ = 1.36 and toluene for yellow; *n*_solvent_ = 1.49). Scale bars, 1 cm.


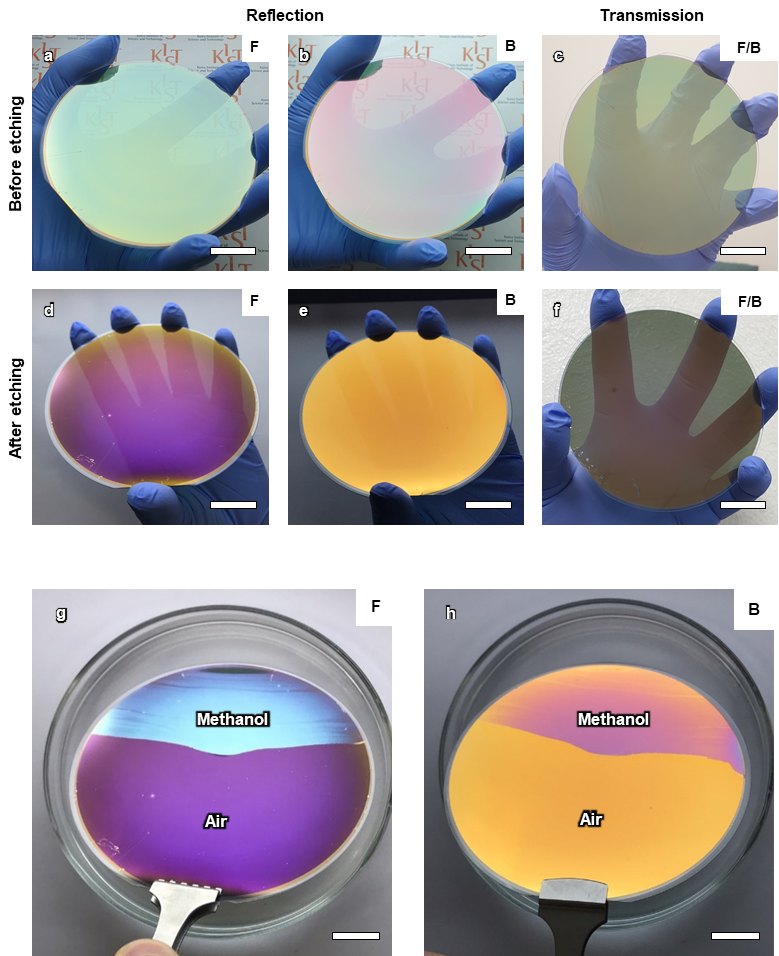


**Fig. S8 Optical Janus effect of BEE at large scale. a-f** Photographs of BEE on s 4-in quartz wafer before- (upper row) and after RIE (lower row), demonstrating its large-area applicability and uniformity. Prior to the RIE process, the optical Janus effect was presented as green/pink colours for F/B (**a**, **b**). After RIE, their reflective colours were drastically changed to purple/yellow for F/B because of the modification of the effective refractive index (RI) in the dielectric layer of BEE (**d**, **e**). While the reflection mode shows unambiguous colour contrast depending on viewing direction, its transmissive colour exhibits invalid colour differences on either side (**c**, **f**). **g**, **h** Observed colours seen from the frontside (**g**) and the backside (**h**) in a 4-in wafer under air and methanol (*f*_Au_ = 0.70), Scale bars, 2 cm.


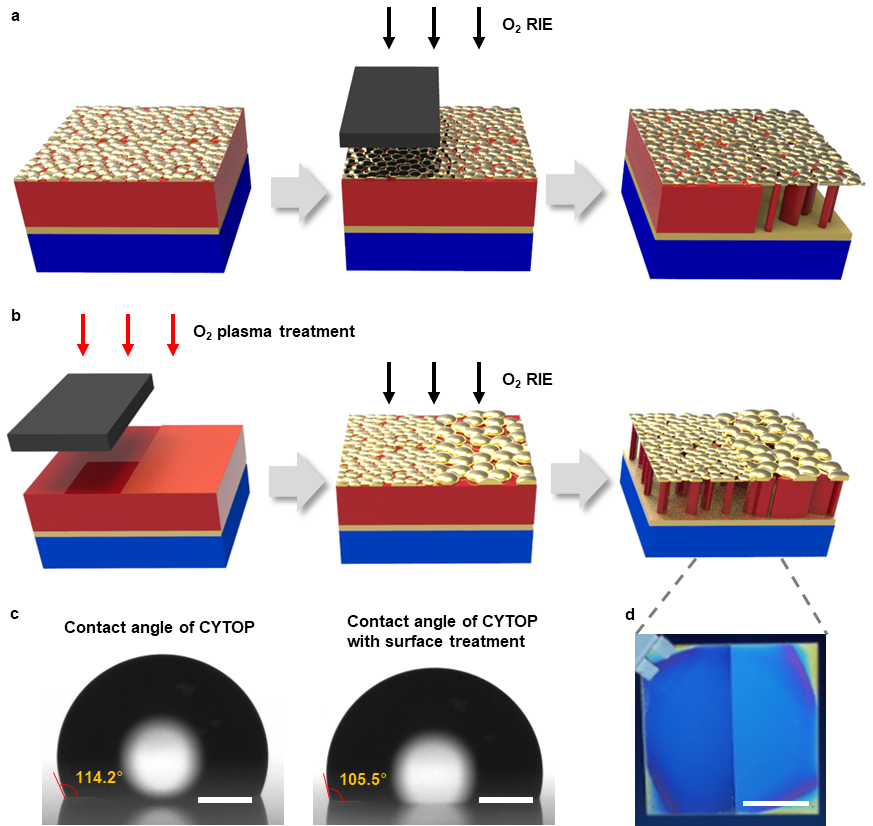


**Fig. S9 Fabrication processes of BEE sample for symmetric/asymmetric message hiding. a**, **b** Schematic illustrations of two types of BEE samples for symmetric- (**a**; site-selective RIE) and asymmetric information encryption (**b**; site-selective plasma treatment). For the symmetric sample, the film geometry of left and right *Regions* on the BEE sample correspond to *Region II* (background) and *Region I* (KIST/KNU message) in the main text, respectively, while *Region I* (background) and *Region III* (KIST message) in the main text can be found in the asymmetric BEE sample. **c** Contact angle images of 3-μL water droplet on CYTOP surface without and with plasma treatment. O_2_ plasma treatment renders the outmost CYTOP surface less hydrophobic by defluorination and incorporation of oxygen-containing groups^2^. **d** Reflective colours on BEE sample etched for 50 s; left area for untreated surface, right for surface-treated area prior to top Au deposition. Scale bar, 1 cm.

**
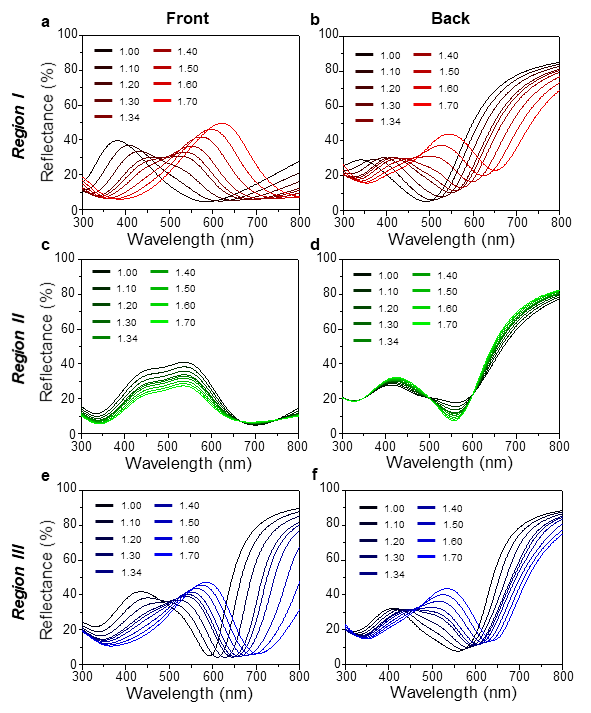
**

**Fig. S10 Simulated reflectance spectra of BEE in each *Region.*** To validate the role of film geometries such as filling fraction of top Au (*f*_Au_) and CYTOP film (*f*_CYTOP_), we conducted simulation and compared the reflectance spectra under various RI values of liquids within each *Region*. **a-f** Reflectance spectra seen from the frontside (**a**, **c**, **e**) and backside (**b**, **c**, **f**) are represented in the physical conditions of the BEE samples introduced in the main text (**a**, **b**; *Region I*, **c**, **d**; *Region II*, and **e**, **f**; *Region III*). The results clearly show that the resonance dip wavelength experiences a red-shift as the BEE samples are exposed to media with higher RI values.

**
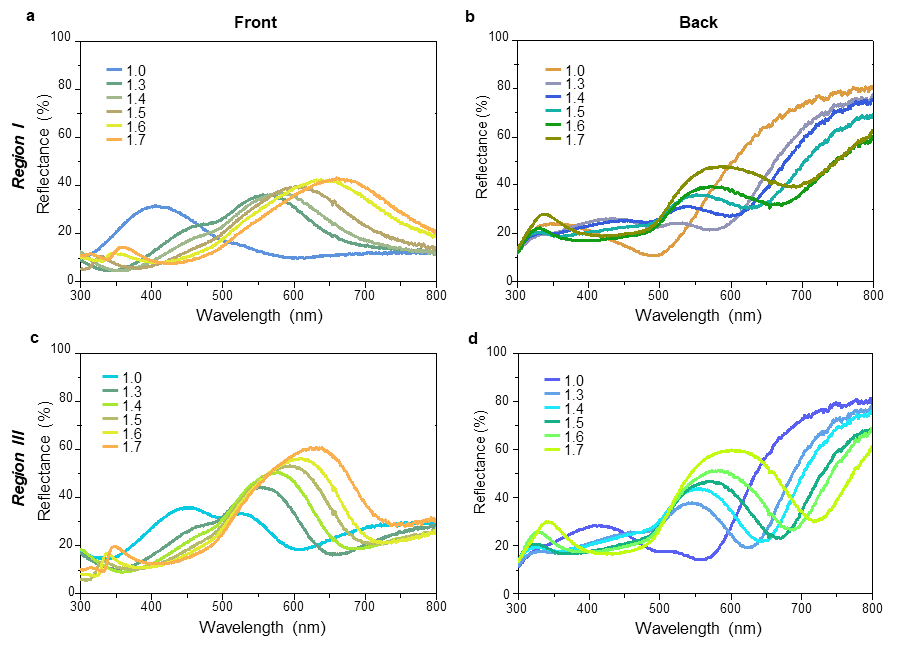
**

**Fig. S11 Reflectance spectra measured from asymmetric message hiding sample depending on mediums with different RIs. a-d** Reflective optical spectra were precisely measured and averaged from more than 5 spots of a BEE sample etched for 50 s consisting of large areas of *Regions I* and *III* (**Fig. S9d**). To examine its RI-dependent optical characteristic, the sample was immersed in various solvents with RIs ranging from 1.3 to 1.7 (1.0 for air) immediately before each measurement. Compared with the broadband absorption of *Region I* (**a**, **b**), *Region III* (**c**, **d**) exhibits sharper FP resonance absorption dips because of its film-like top Au morphology. The greater wavelength shift with respect to the solvent’s RI at *Region I* is attributed to its smaller *f*_CYTOP_ values. This allows dramatic colour changes as a result of the larger amount of material exchange at the air voids when compared with less-RIE-etched areas.

**
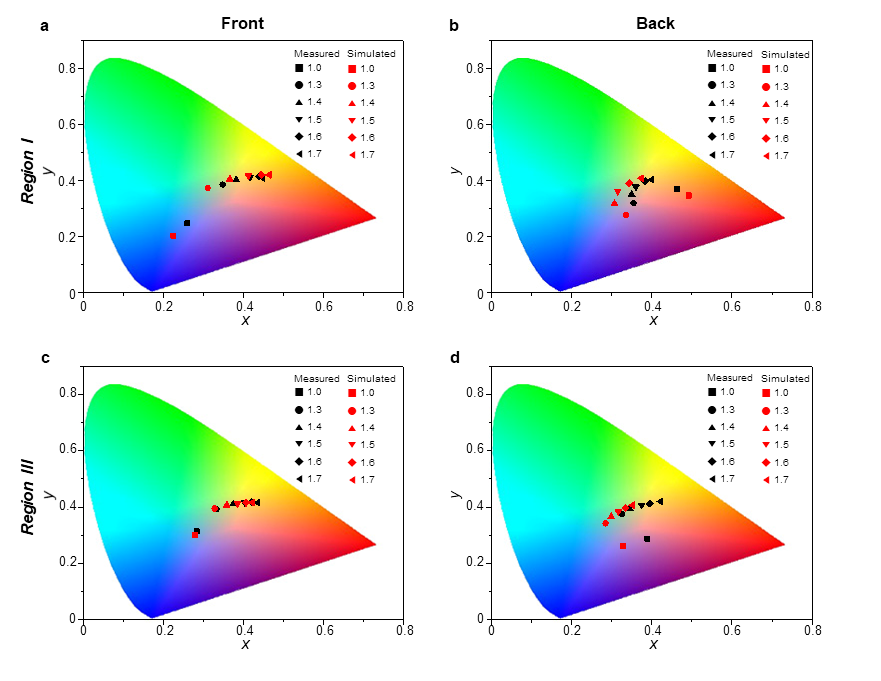
**

**Fig. S12 Reflective colours of BEE on CIE colour map for asymmetric message hiding.** **a-d** To identify the asymmetric colour matching between *Regions I* and *III*, we extracted the *x*, *y* coordinates from the experimental data (see **Fig. S11**) and plotted them on the CIE 1931 colour diagram (black) in comparison with simulated data (red). Despite the slight mismatch between measured and simulated CIE coordinates, plotted marks show a very similar trend on CIE colour maps with respect to the *n*_solvent_. Because of the increased amount of air voids in dielectric films by *f*_CYTOP_, the colour change in *Region I* is more dramatic than that in *Region III*, confirming the higher optical sensitivity (= nm/RIU) to the exposing solvents. In the course of solvent immersion of the BEE sample followed by resultant colour changes, displaying colours in each *Region* (*I* vs. *III*) under particular RI value of solvents eventually become indistinguishable, resulting in colour matching across the boundary (= optical camouflage). Moreover, colour matching solvents across two *Regions* are different side-by-side. The backside displaying colours in each *Region* (*I* and *III*) experiences identical *r*_53_ values, but for multiple term (*r*_51_ - *r*_53_). This results in side-selective colour matching at different RI values of exposing solvents.

Taken together, the various physical properties (i.e., distinct amplitudes and phase shift by *f*_Au_, and optical sensitivity by *f*_CYTOP_) across the Regions lead to side-selective optical camouflage with immersion in different solvents.

To analyse this phenomenon quantitatively, the *x*, *y* coordinates in the CIE colour map are converted into *L*^*^, *a*^*^, *b*^*^ coordinates in CIELAB colour space^3^. The colour difference (Δ*E*^*^) in CIELAB colour space,

$$\Delta E^{*}=\sqrt{(\Delta L^{*})^{2}+(\Delta a^{*})^{2}+(\Delta b^{*})^{2}}$$

becomes a minimum at *n*_solvent_ = 1.4 and 1.6 for the frontside and backside, respectively, which shows good agreement with the simulation results. The slight mismatch of the Δ*E*^*^ minimum condition between experimental and simulated results for frontside observation may be a result of a convolution of technical errors, such as imperfect fabrication, ideal modelling of the effective medium, and errors in the measurement system. Further delicate control of the physical property of comprising BEE films will enhance its message hiding capabilities to the level of complete invisibility across the region boundary.


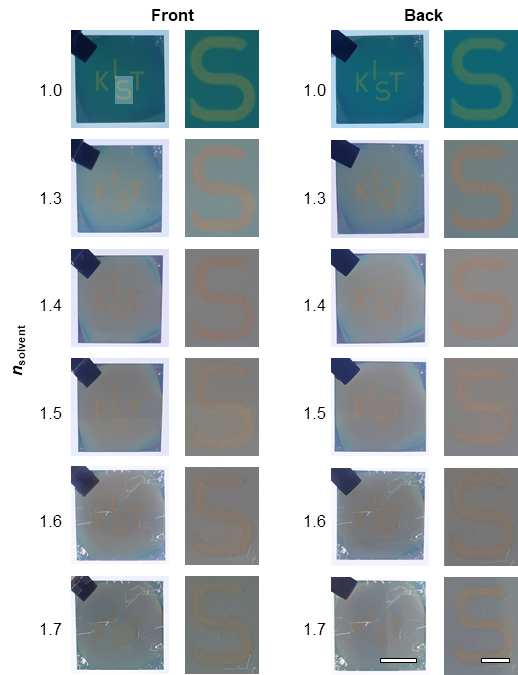


**Fig. S13 Symmetric transmissive colour tuneability of BEE depending on solvent RI.** The message-inscribed BEE, consisting of *Region I* (background) and *Region* *III* (KIST), was exposed to standard RI solvents to verify transmissive colours. The photographs of BEE under different *n*_solvent_ with their enlarged images of the letter 'S’ (white box) show that (i) its transmissive observation exhibits invalid colour difference on either side and (ii) the transmissive colours of *Region I* and *Region* *III* are not completely matched in any solvents. Scale bar, 1 cm (left column) and 2 mm (enlarged right column).


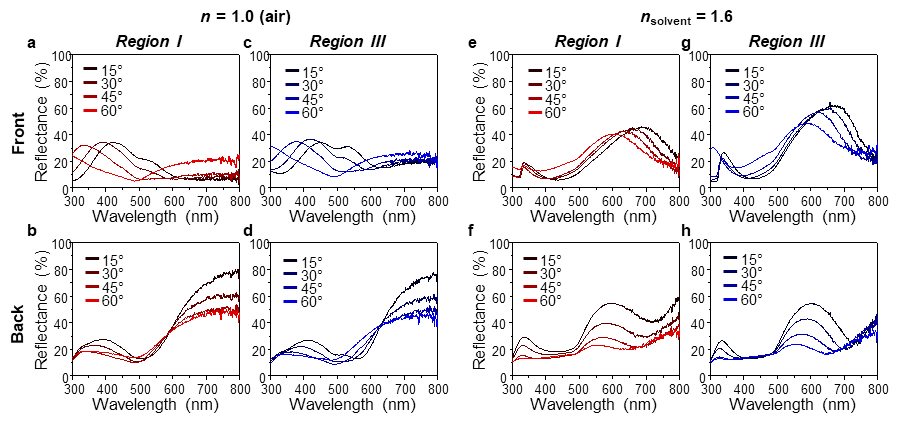


**Fig. S14 Reflectance spectra of asymmetric message hiding BEE measured at various incident angles. a-h** Reflectance spectra of a BEE sample consisting of large areas of *Regions I* and *III* (**Fig. S9d**). The incident angle (*θ*_in_) varies from 15 degrees to 60 degrees. The spectral position of local maximum reflectance (*λ*_max_) was blue-shifted with increasing *θ*_in_ .


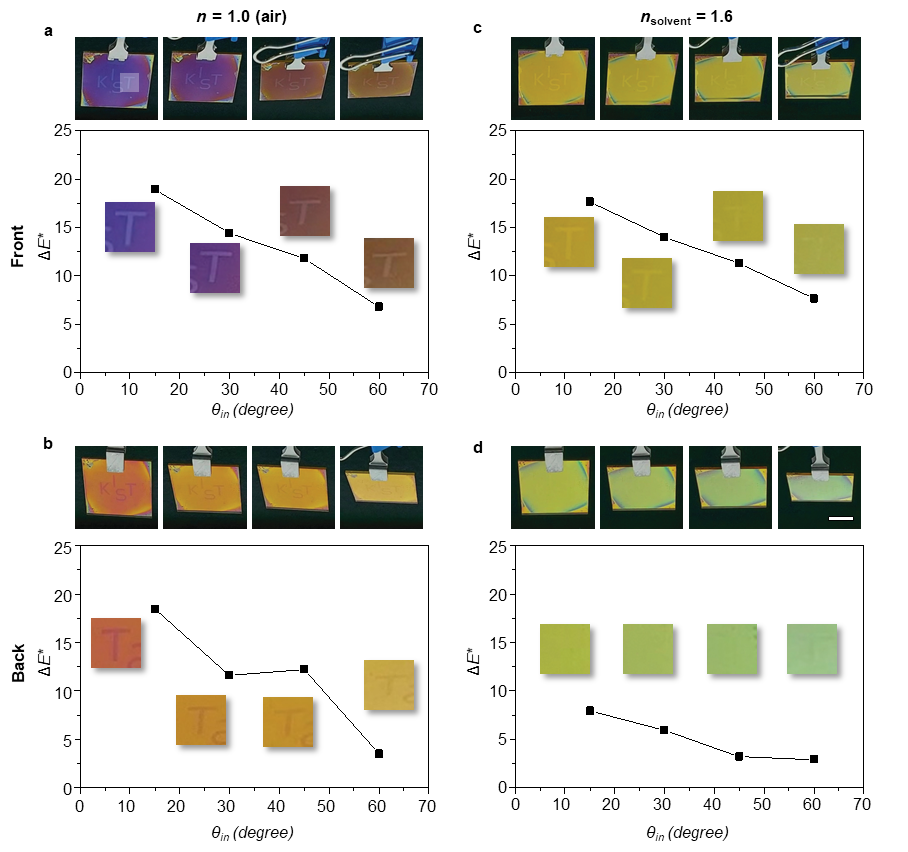


**Fig. S15 Colour tuneability of BEE at various incident angles. a-d** Photographs of asymmetric message hiding BEE and Δ*E*^*^ between *Regions I* and *III* as a function of *θ*_in_. Enlarged images of the letter ‘T’ (white box) are inserted to highlight the colour differences between the two regions. Δ*E*^*^ was obtained from reflectance spectra in **Fig. S14**. Although the blue shift of *λ*_max_ in *Regions I* and *III* occurs identically, the disparity of nanostructures between *Regions I* and *III* results in the variation of Δ*E*^*^ with respect to *θ*_in_. Scale bar, 1 cm.


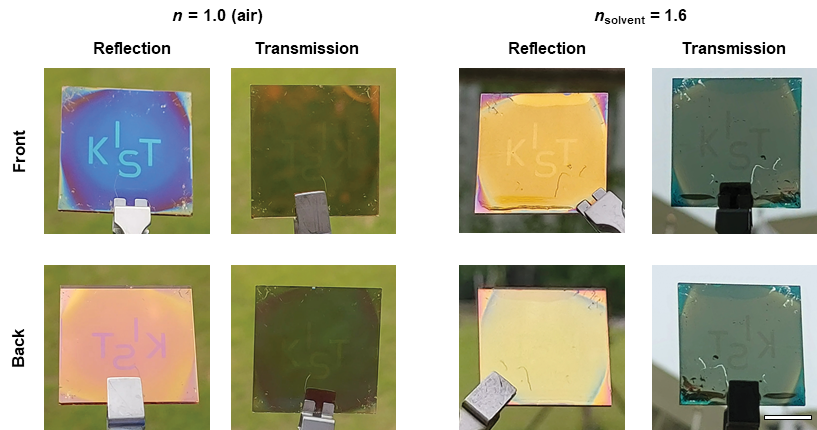


**Fig. S16 Photographs of BEE for asymmetric message hiding under natural sunlight**. The photographs of BEE were taken under diffusive illumination (i.e. natural sunlight). Scale bar, 1 cm.

**References**

1. Kim, H. *et al.* Fourier Modal Method and Its Applications in Computational Nanophotonics. (New York: CRC Press, 2017).

2. Qiu, Y. L. *et al.* Photolithographic patterning of cytop with limited contact angle degradation. *Micromachines* **9**, 509 (2018).

3. Mokrzycki, W. S. & Tatol, M. Colour difference ∆*E* - A survey. *Mach. Graph. Vis. Int. J.* **20**, 383-411 (2011).
